# Factors influencing negative cyber-bystander behavior: A systematic literature review

**DOI:** 10.3389/fpubh.2022.965017

**Published:** 2022-10-03

**Authors:** Sobana Jeyagobi, Shalini Munusamy, Mohammad Rahim Kamaluddin, Abdul Rahman Ahmad Badayai, Jaya Kumar

**Affiliations:** ^1^Centre for Research in Psychology and Human Well-Being, Faculty of Social Sciences and Humanities, Universiti Kebangsaan Malaysia, Bangi, Selangor, Malaysia; ^2^Department of Early Childhood Education, Faculty of Creative Industries, Universiti Tunku Abdul Rahman Sungai Long, Kajang, Selangor, Malaysia; ^3^Department of Physiology, Faculty of Medicine, Universiti Kebangsaan Malaysia, Bangi, Malaysia

**Keywords:** bystander intervention, systematic review, cyberaggression, cyberbullying, cyber-bystander

## Abstract

Cyber-aggression is global epidemic affecting citizens of cyberspace, without regards to physical, geographical and time constraints. Recent research has identified the significant role of cyber-bystanders in exacerbating and de-escalating incidents on cyber-aggression they come across. Additionally, frequent exposure to cyber-aggression is found to have been associated with negative effects on participants of cyber-aggression, ranging from self-esteem problems to mental health disorders such as depression and anxiety, and in the worst cases even suicidal behaviors and ideation. Moreover, past research had also identified that negative bystanders could potentially become aggressors themselves. Therefore, the current review is aimed at uncovering the common themes and factors that drive individuals to resort to negative bystander behavior. Hence, a systematic literature review using the PRISMA framework was carried out, involving articles published between January 2012 to March 2022, on online databases such as SCOPUS, Science Direct, SAGE Journals, Web of Science, and Springer Link. Results obtained through the synthesis of 27 selected articles, were grouped into three categories, namely situational factors, personal factors and social influence. Upon further synthesis of the results, it was noted that many of the factors had interacted with each other. Thus, practical suggestion for prevention and future research would include addressing these interactions in preventative methodologies and research interests.

## Introduction

The introduction of handheld supercomputers such as tablet computers and smartphones has not only made the internet and its affordances increasingly accessible but has also made one's exposure to cyberspace and the digital landscape as inevitable as it is essential. Aside from improving one's quality of life, the increased reliance and almost constant exposure to cyberspace can not only turn into dependence and subsequent addiction, but can also decrease one's quality of life in various aspects [e.g.,: (1–4)], including by exposing individuals to phenomena such as cyber-aggression (5, 6). According to past literature, rates at which cyber-aggression is perpetrated or victimizes an individual, fall between 1 to 41% and 3 to 72%, respectively (7).

Similarly to problematic internet use, it is associated with a host of negative thought patterns and behaviors ranging from mental health problems such as depression and suicide (8), to behavioral problems such as consequent or past involvement in bullying or aggressive behavior (8–11). Despite that, those who experience the fear of missing out (FOMO) tend to refrain from reporting the incident to others due to the fear of losing access to the internet or their phones which keep them connected in ways they desire (12), suggesting that the dependence on the digital world can potentially keep people in a loop of negative experiences.

Given the multifaceted nature of cyberaggression and the need to improve general understanding and intervention efforts associated with it, researchers from various fields have investigated the phenomenon from a variety of angles. These include identifying risk factors associated with cyber-aggressors (8, 10, 13), building standardizable research instruments [e.g.,: (11, 14, 15)], studying the efficacy of intervention strategies (16, 17), improving detection of aggression online (18–20), and more recently, cyber-bystander behavior, among others. The improved understanding of the role of cyber-bystanders in an incident of cyber-aggression has assisted in improving the understanding of the unique mechanism of cyber-aggression, and subsequently, the way it is defined, as well as the way intervention efforts are approached.

For instance, the presence and influence of cyber-bystanders are one of the reasons which prompted researchers to call for cyber-aggression to be distinguished from face-to-face aggression, defining it simply as being an act that is carried out using Information and Communications Technology (ICT), with the intent to cause harm to an individual (or individuals) who would have rather avoided it, omitting factors such as repetition and power imbalance which characterize traditional bullying or aggression (21, 22). This not only acknowledges the significant impact of other factors that mediate and exacerbate or merely influence cyber-aggression such as the presence and actions of cyber-bystanders but also allows for the inclusion of other singular but harmful acts such as happy slapping and outing (22, 23).

Cyber-bystanders, in simple terms, make up the audience who not only witness incidents of cyber-aggression but also have the capacity to either escalate or de-escalate the severity of the incident they have witnessed through their own response or even lack of response (21, 22). While many cyber-bystanders do utilize the opportunity to intervene positively and defend and/or comfort the victim, or even confront the aggressor constructively in order to de-escalate the situation, a non-negligible number of individuals engage in bystander behavior which reinforces the act of aggression against the victim or may even become hostile toward the aggressors (24). Moreover, cyber-bystanders are also at risk of becoming primary aggressors themselves (24).

Additionally, past research has identified that their impact on cyber-victims is no different than that of a primary aggressor. For instance, victims become warier of their social environment or experience fear and worry about being revictimized and having to revisit the incident and the pain it elicits whenever someone shows support for the aggressor by interacting with the victimizing post (12). Additionally, young people express that they do not receive the necessary support from their environment and admit to being unequipped to aid their peers manage and resolve these experiences (12). Hence, it is no wonder that feelings of loneliness, mistrust, embarrassment, fear, sadness and helplessness, and depressive symptoms as well as suicidal ideation, were associated with these revictimized individuals (25).

Furthermore, having to face incidents of cyber-aggression even as a bystander, was found to be associated with depression and/or anxiety due to the fear of being victimized, feeling empathic concern for the victimized individuals followed by the helplessness that results from not knowing how to navigate through the situation (26). In addition to that, frequent and long-term exposure to cyber-aggression on the internet could also result in individuals having lesser levels of empathic responses toward distressed individuals over time (27), and if bystanders were to perceive cyber-aggression as being a norm or expectation within their social circle, they are more likely to reinforce such acts (28).

Therein lies the importance of addressing what drives the behavior of bystanders, particularly those individuals who choose to engage in behavior that explicitly exacerbates a cyber-aggressive act, in addition to individuals whose passive behavior can act as silent approval of an instance of cyber-aggression. Therefore, the aim of the current review is to identify and synthesize results from past studies which have pinpointed the factors that influence an individual's decision to resort to negative bystander behavior. Additionally, the types and the frequencies of negative bystander behaviors addressed in the selected studies will also be highlighted for context and a better understanding of the phenomenon.

## Methods

### Identification of literature

Literature was sourced using five different online databases, namely Scopus, Science Direct, Web of Science, Springer Link, and SAGE Journals. The search was conducted during the last week of February 2022 up to mid-March 2022, targeting articles published in the past decade and throughout the search period.

Keywords to be used were identified by reviewing past literature addressing negative cyber-bystander behavior—words associated with the term “bystander” and the various forms of negative bystander behavior were sourced. The search resulted in the finding that cyber bystander was used interchangeably with the words “cyber observer” and “cyber witness”, and the types of negative bystander behavior can be summarized as being behaviors that reinforce the aggressor or act of aggression, aggressive behaviors, or passivity. Additionally, before identifying the keyword string to be used, the search features of the selected online literature databases were investigated. Special symbols to promote truncation of used keywords were omitted as they were already built into the database search engines.

The final string of keywords used was “((cyber bystander) OR (cyber witness) OR (cyber observer)) AND (aggressive OR passive OR reinforce)”, whereby the keyword string was divided into two halves, containing the synonyms of “cyber-bystander” in the first half and keywords associated with negative bystander behavior for specification and focus in the second half.

### Eligibility and screening

Literature was screened in multiple stages, beginning from the inspection of the title and abstract to select suitable articles from the databases, followed by the close examination of the full-text of the article to determine whether they qualify to be included in the review. Inclusion and exclusion criteria were set as parameters to aid these processes, to ensure that a coherent set of articles are selected to be included in the review, allowing the researcher to fulfill the purpose of the study as accurately as possible.

#### Eligibility

Firstly, in order to ease the process of reviewing the articles, and to avoid misinterpretation of the contents of the articles due to flaws in translation, the articles screened were limited to those written in the English language. Secondly, a timeline spanning a decade, from 2012 to the current year, was set, taking into account the development of the technological scene which may have differed too vastly in the previous decade given how rapidly technology has evolved, possibly affecting the findings regarding individuals' behavior online. In addition to that, only research articles that discuss and elaborate on negative cyber-bystander behavior, which includes behaviors such as assisting the bully, aggressive intervention, and even the absence of intervention entirely, were included.

In the current review, negative bystander behavior was characterized as any response to cyberbullying which encourages bullying, including the absence of intervention as it is viewed as silent approval and reinforcement of the act of bullying, and “aggressive defending” through which a bystander defends the victim *via* acts of aggression against the bully. Other such acts include reinforcing the bully by assisting them, encouraging them through laughing along or sharing the content with others, and the like. This would indefinitely exclude articles which only address positive and constructive bystander behavior which express support toward the victim and disapproval of cyberaggression without the use of excessive aggression.

Additionally, articles which do not clearly identify the relationship between identified factors and negative bystander behavior and approach the topic through the lens positive bystander behavior will also be excluded to avoid misinterpretation of the results. The criteria are better presented in Table 1.

**Table 1:** Screening criteria.

**Criteria**	**Inclusion**	**Exclusion**
Timeline	Between 2012 and 2022	Before 2012
Language	English	Languages other than English
Type of articles	Research articles	Articles other than research articles (e.g.,: review, conference proceedings, books, etc)
Content	Factors influencing negative bystander behavior and decision making in cyberbullying situations	Does not address factors influencing negative bystander behavior and decision making in cyberbullying situations (e.g.,: articles that discuss traditional bullying, positive bystander behavior, etc)

#### Literature search and screening

The literature search was split into two sections, whereby the first part of the search involved using search engines of literature databases to recall articles relevant to the keyword string input, and the second part involved a backwards and forwards search using relevant articles.

**Figure 1:**
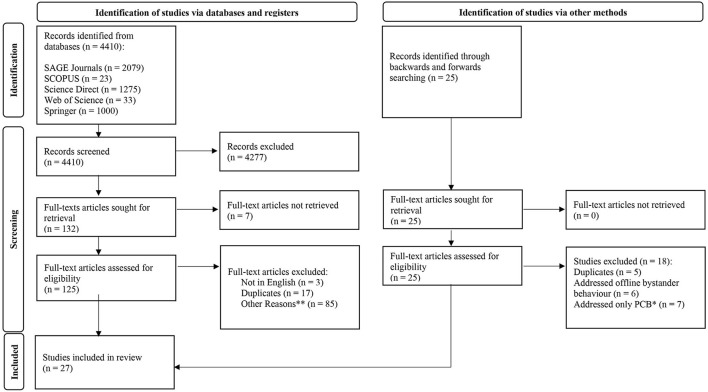
PRISMA flow-diagram. Source: Page et al. (29).

Through the preliminary literature search using the literature databases, 4,410 articles were identified and were screened based on their abstract and title. This preliminary screening resulted in 132 articles being identified and selected for further screening. On the other hand, as for the backwards and forwards search, a randomly selected article published in 2020 and 2013, respectively, were used to fill timeline gaps. The literature database “*Web of Science*” was used for these processes, which resulted in 25 most relevant articles being identified through the screening of their title and abstract alone. Next, upon the removal of articles that could not be accessed, were not written in English and were duplicates, authors were left with 116 articles to be screened. These articles were screened based on their full-texts to determine whether or not they met the inclusion criteria set. This resulted in 27 relevant articles being selected as the most suitable to be included in systematic review and fulfill the research objective of identifying the factors associated with negative bystander behavior. The process is presented in the PRISMA (Preferred Reporting Items for Systematic Reviews) flow-diagram (Figure 1).

Selected articles were then screened for author names, age range of participants, study design, types and prevalence rates of negative cyber-bystander behavior, and lastly the factors that contribute to negative cyber-bystander behavior. The data extracted are presented in Table 2 and discussed in the following section.

**Table 2:** Articles included in the study.

**References**	**Age range/mean age (years)**	**Study design**	**Type/prevalence of negative bystander behavior**	**Factors influencing negative bystander behavior and decision making & their prevalence**
Balakrishnan (48)	21.0	Cross sectional quantitative survey	61.5% defended the victims, 40.1% didn't do anything, 17% supported the bully	Safety, believed that it was not their problem
Barlinska et al. (42)	11–18	Experimental	Passive bystander behavior, reinforcer	Previous experience as cyber-aggressor, private nature of act
Bastiaensens et al. (28)	15.78	Cross sectional data analysis (data obtained from 4th wave of Longitudinal study)	5% joined the bully	Perceptions pertaining to peers' approval of cyber-aggression, Frequency of experience as a cyber-aggressor
Bastiaensens et al. (31)	13.29	Experimental quantitative survey	Reinforce bully by; sharing it with others to make fun of the victim, Telling the bully that you find it funny, Doing something similar	Behavior of good friends when faced with a bullying situation
Bauman et al. (50)	13.69	Exploratory analyses	10.55% did not intervene	51% Didn't know what to do, 38% Not my business, 38% I am too shy, 28% Didn't want to be a “snitch”, 28% Afraid of being bullied, 16% The bully is popular, 16% It wasn't that serious, 12% don't care, 11% didn't like the person being bullied, 11% I thought the victim could take care of it themselves, 11% “Others didn't do anything so why should I?”, 8% The bully is my friend.
Bussey et al.. (54)	11–15	Quantitative Survey	Aggressive defending: Saying mean things about the bully, Threatening the bully, Asking the bully to “back off”	Low defending self-efficacy, moral disengagement
Chan et al. (49)	Students: 13–17 years old Counselors: 29–57 years old	Hermeneutic phenomenological Study	Passive bystander behavior, reinforcing aggressors	Ignorance about cyberbullying and its effect, moral disengagement, fear, severity of incident, did not want to be involved
Cleemput et al. (43)	9–16	Cross sectional Quantitative survey (contained open ended questions)	35.2% Bystanders: Passive bystanders, Assistants	Age, empathy, past experience as a cyber-aggressor or witness, 31.8% Fear of retaliation, 30.6% lack of skill, 15.4% lack of self-efficacy, 13.0% displacement of responsibility, 49.0% believed that it was not their responsibility, difficulty assessing the situation, asynchronicity
DeSmet et al. (52)	13.61	Cross sectional quantitative survey (contained open ended questions)	55% did nothing, 41.3% deleted support for their victim, 14.6% laughed without letting anyone notice, 10.4% laughed while letting the bully notice (reinforcing the bully), 1.8% forwarded the content to someone else	Intentions to resort to negative bystander behavior, positive attitudes toward negative bystander behavior, higher expectations that negative bystander behavior would lead to personal gains, low problem-solving skill, lower empathetic skills, cognitive restructuring, lack of supportive factors that encourage positive bystander behavior
Erreygers et al. (41)	12.6	Cross sectional quantitative survey	53.6% did nothing, 4.6% joined the bully	Low empathy, high impulsivity, age
Gahagan et al. (55)	21	Quantitative survey (with qualitatively analyzed open ended questions)	Passive bystander behavior	Diffusion of responsibility, severity of bullying, relationship with victim
Koehler and Weber (61)	21.3	Experimental	Passive bystander behavior	Severity of the incident
Levy (65)	16.31	Cross sectional quantitative survey	45% Bystanders in total, 18.2% passive bystanders, 10.8% aggressor-supporters	Parental monitoring
Luo and Bussey (57)	12–15	Cross sectional quantitative survey	Aggressive defending: Threaten bully, Say mean things about the bully, Put the bully's information online, Make up rumors about the bully	Greater general and contextual moral disengagement
Machackova et al. (46)	15.1	Cross sectional quantitative survey	12% were passive bystanders	Relationship with the victim, gender, high self-esteem
Machackova and Pfetsch (53)	14.99	Cross sectional quantitative survey	Join the bully in bullying	High normative beliefs that aggression is an appropriate response to provocation
Moxey and Bussey (69)	13–16	Cross sectional quantitative survey	Aggressive defending	Past-experience as cyber-aggressor
Olenik-Shemesh et al. (51)	12.87	Cross sectional quantitative survey	55.4% passive bystander behavior	16.8% fear, 38.6% “Not my business”
Panumaporn et al. (44)	14.97	Cross sectional quantitative survey	26.3% Assistants, 28% passive bystander	Past-experience as cyber-aggressor, relationship with victims and/or other participants
Patterson et al. (63)	13–16	Vignette interview	Assistants, passive bystander behavior	Interpretation of situation, gender of protagonist, relationship with participants, severity of the incident
Patterson et al. (59)	13–16	Qualitative interview	Passive bystander	Absence of physical danger, lack of rules and absence of figures of authority online
Schultze-Krumbholz et al. (47)	13.44	Cross sectional quantitative survey	8.1% assistants, 9.5% aggressive defenders, 28.4% outsiders	High levels of reactive aggression, experience as cyber-aggressor and/or cyber-victim, low levels of socio emotional competencies, lack of empathy
Schultze-krumbholz et al. (40)	11–17	Cross sectional quantitative survey	Assistant	Less positive peer interactions in class
Song and Oh (64)	16.5	Cross sectional quantitative survey	60.7% outsiders, 5.4% reinforcers, 3.3% assistants	Positive relationship with the bullies
Tong (58)	8–16	Experimental vignette	Passive bystander behavior	Moral disengagement, low moral responsibility, past experience as bully-victims
Wang et al. (45)	18+	Cross sectional quantitative survey	Passive bystander behavior	Gender, age
You and Lee (60)	25–51	Experimental	Passive bystander behavior	Anonymity, number of bystanders

Some of the reasons articles were excluded include the fact that they did not discuss negative cyber-bystander behavior, and discussed positive cyber-bystander behavior [e.g.,: (30–33)], addressed bystander behavior in offline settings [e.g.,: (34, 35)], addressed cyberaggression rather than reinforcement of the act through negative cyber-bystander behavior [e.g.,: (36–38)], how people protect themselves [e.g.,: (39)], and the like instead.

## Results

The analysis of included studies reveal that there are four different types of negative cyber-bystanders—passive bystanders, assistants, reinforcers and aggressive defenders. These bystanders, remain passive, join in on the aggression, show support for the aggressor, and aggress against the aggressor, respectively. “Showing support for the aggressor” within this review, was found to be limited to behaviors such as sharing the incident with others with the intent of making fun of the victim, laughing at the incident, telling the aggressor that they found the situation funny, and cheering on the bully. On the other hand, “aggressive defending” included behaviors such as threatening the aggressor, spreading rumors about the aggressor, or releasing their private information online, saying mean things about the aggressor and lastly, asking the aggressor to “back off”.

Out of the twenty seven studies included in this review, only eleven studies reported prevalence rates of negative cyber-bystander behavior, and these rates fell between the ranges of 1.8–55.4%, depending on the type of behavior measured. The most common negative cyber-bystander behavior was passive bystander behavior, with prevalence rates ranging from 10.55 to 55.4%, followed by reinforcers, assistants, and aggressive defenders with rates as low as 1.8% and a maximum of 26.3% overall. While the prevalence of the latter types of negative cyber-bystander behaviors are much less common, they have a more direct effect on the victim, and the severity and/or direction of an incident.

In terms of the factors that were associated with these behaviors on the other hand, the analysis of the results indicates that the most studied variables were what the current review categorizes as “personal factors”, and the least commonly addressed variables were associated with social influences. Moreover, there are a noteworthy number of studies which both, studied and loosely addressed “mediating” variables to explain the motivation behind negative cyber-bystander behaviors. Additionally, many studies had discussed certain parts of their findings (i.e.,: the relationship between the variables studied and cyber-bystander behavior) in relation to constructive cyber-bystander behavior without clarifying how or if said variables influence or are associated with negative cyber-bystander behaviors. Hence, only certain variables from these studies can be reported within the current review, and are reported alongside other details of the study in Table 2. Following that, the factors associated with negative cyber-bystander behavior which were identified through the review process were then grouped into three categories, namely (a) personal factors, (b) situational factors, and (c) social influence, and are presented in Table 3, and discussed in the sections below.

**Table 3:** Factors influencing negative cyber-bystander behavior identified in the current review.

**Categorization**	**Factors**
Personal factors	**Demographic background** •Age •Gender
	**Beliefs, norms and outcome expectations** •Perceived rewards •Perceived harm •Positive attitudes toward negative cyber-bystander behavior
	**Skills and self-efficacy** •Lack of problem-solving skills •Low socio-emotional skills •Low social self-efficacy •Perceived lack of defending skills
	Low empathy
	Aggressive tendencies
	Moral disengagement and responsibility
Situation factors	**Characteristics of cyberspace and computer mediated communication** •Lack of physical aggression •Lack of clear rules •Lack of guidance online from authority figures •Delayed exposure to the event
	Privacy of the incident
	Anonymity
	**Interpretation of the incident** •Perception that the event is not severe enough to warrant intervention •Perception that the incident is banter •Feelings of enjoyment when witnessing the event •Belief that the victim can handle the situation •Uncertain of the nature of the situation
	**Other participants** •Behavior of other cyber-bystanders •Presence of other cyber-bystanders •Gender of protagonist
Social influence	Relationship with other participants
	Popularity of aggressor
	Perceived peer response
	Parental monitoring
	**School environment** •Lower levels of positive peer relationship •Higher number of aggressors in class

### Personal factors

These factors address qualities unique to an individual, such as their demographic backgrounds, their past experiences, levels and usage of moral disengagement strategies, empathy levels, skills and beliefs pertaining the efficacy of their capacity.

#### Demographic background

While various demographic factors were addressed, age and gender were the most commonly studied across the articles included in this review and were the only demographic factors identified as being significantly associated with negative cyber-bystander behavior, with some exceptions which provided contradicting results by Schultze-Krumbholz et al. (40), Erreygers et al. (41), and Barlinska et al. (42).

With regards to age, it was noticed that older individuals expressed more reluctance to intervene (41), and were more likely to remain passive as a bystander (43) or join in on the aggression (43, 44). The lack of constructive intervention and higher negative cyber-bystander behavior among older individuals in the sample populations were rationalized using low empathy scores (45), the increase in anti-social tendencies in older adolescents (44) and social influence such as the fear of judgment by peers (41).

Gender differences reported by the studies included in the review, on the other hand, seemed to consistently indicate that male individuals were more prone to negative cyber-bystander behavior, in comparison to their counterparts. Not only was it identified that boys had a higher likelihood of being passive (45, 46) and possessing higher behavioral intention to reinforce the aggressor (31), being male could also significantly predict an individual's involvement as an assistant (47). However, variables such as moral disengagement strategies which distort the consequences of their behaviors, as well as perceived severity were found to moderate the relationship between gender and pro-aggressor behaviors in the study by Schultze-Krumbholz et al. (47), suggesting that the relationship between gender and negative cyber-bystander behavior might not be very straight-forward and will require further analysis.

#### Past experience as an aggressor or victim

Unsurprisingly, studies found that individuals with past experience as an aggressor were more likely to be associated with negative cyber-bystander behavior (28, 42–44). This was true for both traditional aggressors who engaged in face-to-face aggression, as well as cyber-aggressors who were bullies in cyberspace (43). Panumaporn et al. (44) elaborated on these results, stating that individuals who were bullies tend to hold positive beliefs pertaining to the use of aggression and that this attitude is likely to be reflected in their behavior as cyber-bystanders as well. Moreover, individuals who were aggressors did not feel pressured into joining in on the aggression as cyber-bystanders (28). On the other hand, it was identified that aggressive defenders were also more likely to have had experiences of being cyber-victims in the past, in comparison to their peers who engaged in more prosocial behaviors (47).

#### Beliefs, norms, and outcome expectations

Seven different studies identified differing normative believes, outcome expectations and attitudes pertaining to cyber-aggression or bystander behavior. Put simply, the findings of these studies indicate that individuals tend to engage in behaviors that they perceive would result in either a reward or the avoidance of harm. For example, individuals stated that they remained passive for safety reasons (48) or the fear of being victimized in the process of defending a victim (43, 49–51). On the other hand, DeSmet et al. (52) found that individuals were also prone to remaining passive or reinforcing the aggressor if they expected to be rewarded with a rise in social status, the possibility to gain new friends, for self-protection or if they held positive beliefs about their choice of response as a bystander. Additionally, it was also noticed that individuals who believed that aggression was a valid response to provocation, as well as those who had lower levels of defending normative beliefs were moe likely to resort to pro-bullying behavior that reinforces aggression (53).

#### Skills and self-efficacy

Whether or not individuals possessed the skill or the belief that they were capable of effectively handling an incident of cyber-aggression as a bystander, was found to be associated with negative bystander behavior. In essence, individuals who lacked self-efficacy or the skills to intervene, had a higher tendency to become negative bystanders (43). While participants in the study by Bauman et al. (50) simply stated that the “did not know what to do” in response to why they remained passive, more specific factors such as the lack of defending self-efficacy (54), social self-efficacy and knowledge (55), as well as low socio-emotional skills (47), self-reliance, and problem-solving strategies) were linked to the increased tendency to resort to negative cyber-bystander behavior. Van Cleemput et al. (43) suggested that the lack of control individuals have over an incident of cyber-aggression, such as the inability to prevent something from going viral given the public nature of cyber-space as well as the speed through which information spreads digitally, could contribute to the perceived lack of skills (i.e.,: low self-efficacy) among individuals. It is important to note, however, that the adolescents in the study by Bussey et al. (54) neither remained passive nor reinforced the aggressor, but instead resorted to aggressive defending styles by redirecting the aggression toward the aggressor when they lacked the self-efficacy to defend individuals in more constructive ways.

#### Empathy

Cross-sectional studies show that lower levels of empathy was able to predict individuals' negative bystander behavior (43, 47). Additionally, an empathic reaction was found to be the only differentiating factor between bystanders who respond in supportive ways and those who chose to remain passive (46). Lastly, a noteworthy finding by Machackova et al. (46) indicates that it's likely that empathic reactions could be reliant on contextual factors that might play a more crucial role in determining the final response.

#### Aggressive tendencies

Without surprise, studies found that more aggressive individuals had a higher tendency to assist aggressors (47). Schultze-Krumbholz et al. (47) highlighted reactive aggression in particular and suggested that the behavior is likely influenced by impulsivity and identified this group of individuals as being at a higher risk of becoming aggressors, in comparison to their peers.

#### Moral disengagement and responsibility

Moral disengagement was found to be one of the more prominent theme and variable in this review, with seven different studies highlighting its link to negative bystander behavior. It is defined as a mechanism that allows an individual to reconstruct their beliefs about negative and harmful behaviors by either minimizing or distorting the consequences of said behaviors, or shifting the blame and/or responsibility away from themselves or onto others, allowing them to justify engaging in such behaviors (56). The results presented by the studies indicate that individuals who were more morally disengaged were also more prone to engaging in aggressive defending (54, 57), as well as positive bystanding (49, 58). This is likely because moral disengagement processes provide individuals with the tools to justify their behavior (49), even in the event that they behave in ways that contradict their own values (58).

Van Cleemput et al. (43) who studied the influence of various aspects moral disengagement mechanisms identified that diffusion of responsibility, attribution of blame, and distortion of consequences as well as displacement of responsibility as being factors that are positively linked to negative cyber-bystander behavior. Similar patterns can be found in other studies as well, where individuals who had low levels of moral responsibility (58) or believed that it was “not their problem” (48, 50, 51, 55) were more likely to be negative bystanders. DeSmet et al. (52) on the other hand, found contradicting evidence pertaining the mechanism of attribution of blame, whereby lower victim blaming tendencies were associated with passive bystander behavior, suggesting that it is possible that individuals were behaving in ways that go against their own beliefs.

### Situational factors

#### Characteristics of cyberspace and computer mediated communication

Three different characteristics of computer mediated communication (CMC) were identified through this study. The first being the absence of physical aggression which diminishes the severity of the event in the eyes of cyber-bystanders (59). Secondly, student's suggested that the lack of rules and authority figures present in online spaces, to provide guidance, led them to remain passive when they witnessed incidents of cyber-aggression (59). Lastly, it was noticed that the increase in tendency to be a passive bystander was contributed by the bystanders' delayed exposure to the incident of cyber-aggression (43).

#### Privacy

Participants in a study by Barlinska et al. (42) were found to be more likely to remain passive as a bystander when the incident they had witnessed was private in the nature. The researchers theorized that it was the result of the lack of social pressure to conform to social norms, which is present in situations where there is a large audience.

#### Anonymity

In the study by You and Lee (60), it was identified that the intention to intervene was more influenced by their own anonymity or lack thereof, rather than the number of cyber-bystanders present.

#### Interpretation of the incident

In most of the reported findings addressed in the studies included in the current review, it was noticed that individuals were more likely to remain passive if they perceived a situation as being not severe enough to require intervention (49, 50, 55, 59, 61). This falls in line with the Bystander Theory by Latane and Darley (62), which states that individuals need to perceive the situation as being an emergency that requires their intervention in order to engage in proactive and constructive bystander behavior. Additionally, it was also identified that individuals remained passive if they assumed that the victim had the capacity to handle the situation themselves, or if they found enjoyment in witnessing the incident (50)—it seems reasonable to assume that that this would have made a situation seem less like an emergency that requires their intervention.

In addition to the perceptions about the severity of the incident, two studies found that bystanders also remained passive when they experienced difficulty in interpreting the nature of the incident they are witnessing (43, 63) and/or were uncertain about who was responsible for the incident (43). On the other hand, they were more likely to engage in behaviors that reinforce the aggression and aggressor if they believed that the event they were witnessing was a joke between parties involved (63).

#### Other participants

Cyber-bystanders' behavior was found to be influenced by not just the behavior of other bystanders (31, 49, 50) but also the number of bystanders present (60, 64), as well as the gender of the protagonist (63) involved in an incident of cyber-aggression. Through this review, it was noticed that bystanders were likely to reinforce aggressor (31) or remain passive (50), if other bystanders, especially close friends were to engage in such behaviors (31). Additionally, the “bystander effect” which states that individuals are less likely to intervene in the presence of a large audience, was present in reports by Song and Oh (64) as well as You and Lee (60). However, it was reported that passive bystander behavior was possible even in the absence of other bystanders, in the event that these bystanders had a positive relationship with the aggressor(s), likely to preserve their relationship with the aggressor (64), suggesting that there are other important contextual factors that need to be taken into account when addressing the number of bystanders and its relationship with the behavior of bystanders.

Lastly, a lone study by Patterson et al. (63), in which adolescents were allowed to freely state their reasons for passive behavior, it was identified that individuals did not want to intervene in situations where the protagonists were female as they believed that the situation was less controllable than when the protagonists were male.

### Social influence

#### Relationship with other participants

As addressed in an above-mentioned section, bystanders' behavior was also found to be influenced by their relationship with the participants involved in an incident they had witnessed. For example, if they were friends with aggressor, they were more likely to reinforce or ignore the incident and disregard the plight of the victim (50, 64) in order to maintain their relationship with the aggressor (64). However, Song and Oh (64) clarified that this behavior was context dependent and relied on the absence of other bystanders, as the presence of other bystanders would lead to defending behaviors.

Additionally, bystanders were also found to remain passive when they were not closely acquainted with or had a bad relationship with the victim (46, 50, 55), or in the event that they did not have a close relationship with any participant involved in the incident of cyber-aggression they witnessed (44, 63). It could be because individuals perceived levels of responsibility based on the closeness of their relationship with the victim (55)—the closer they were, the more responsibility they had to intervene and defend the victim, and vice versa (44). It could possibly be explained by how people tend to view individuals from their in-group and their out-group as most people tend to prioritize their in-group (i.e.,: individuals they identify with, and are close to) in comparison to those who are more distant to them (44). Machackova et al. (46) on the other hand, theorized that an individual's relationship with the victim could influence their perception of the severity of the event they are witnessing, meaning the closer they were to the victim, the more severe they would perceive an incident to be. According to the “bystander effect” theory and the bystander intervention model, a lower perception of severity would lead to passivity in the face of aggression as a bystander.

#### Popularity of aggressor

The popularity of the aggressor was stated as a reason why 16% of the bystanders in the study by Bauman et al. (50) had refrained from intervening and had chosen to remain as passive bystanders. It could be that going against a popular individual who holds a higher social status costs a lot more than individuals are willing to deal with.

#### Perceived peer response

In the event that individuals held the perception that their peer would support or expect them to join in on the bullying, they were more likely to join in on the bullying and reinforce the aggression against the victim, to maintain their relationships and social status (28, 31).

#### Parental monitoring

Only Levy (65) addressed the influence of parental monitoring. It was noticed that higher aggressor-supporter scores were positively associated with the behavior of reinforcing and supporting aggressors. However, it was not expressed as a causal relationship and authors suggest that restrictions could be the consequence of aggressor-supporter behaviors and questioned the efficacy of such measures if it were the case.

#### School environment

Schultze-Krumbholz et al. (40) found that lower levels of positive peer interactions in class was associated with assistant behavior cyber-aggression situations. Additionally, they also stated that classrooms that contained a higher number of offline-aggressors tend to promote more negative cyber-bystander behavior—likely due to social norms and pressure.

## Discussion

The acknowledgment of the influence of cyber-bystanders in influencing incidents of cyber-aggression has allowed for a more complete understanding of the mechanism of cyber-aggression, which in turn allows for the identification of crucial risk factors that contribute to the reinforcement and/or prevalence of the phenomenon. While these individuals known as negative cyber-bystanders, in contrast to the more constructive cyber-bystanders, have been studied for a while now, the data obtained are scattered and lacking. Hence the current review aims to compile, present and discuss existing findings from journal articles published in the past decade, to provide a more coherent look at the data, and highlight potential findings of interest that may aid in the identification of future research questions. The discussion will be broken into several sections which will discuss the factors associated with negative cyber-bystander behavior, as well as directions for future studies and limitations of the current review.

### Factors associated with negative cyber-bystander behavior

Based on the review of the results and discussions produced by the studies included, it appears evident that negative cyber-bystander behavior is the result of the interaction of multiple factors, and that mediating and moderating factors should be of focus and rigorously studied to better understand the phenomenon. Despite the complexity of the associations between the variables of interest, several notable themes were identified. Firstly, the review indicates that the Bystander Intervention Model introduced by Latane and Darley in 1970 (61) was a relatively simple yet useful model that identified noteworthy precursors to negative cyber-bystander behavior.

Latane and Darley's (62) Bystander Intervention Model posits that (a) the perception of the severity of an incident and need for intervention, (b) accepting responsibility to intervene and (c) having the capacity to intervene were important precursors to bystander behavior (61). The findings identified through the review echoed this, as those who (a) minimized the severity of the event (49, 50, 55, 59, 61), (b) did not think that it was their responsibility to intervene (43, 48, 50, 51, 55), and those who had neither the necessary skills and knowledge (43, 50) nor the self-efficacy to intervene (54), had consequently resorted to negative cyber-bystander behavior.

The need for intervention appears to be assessed in two ways; based on (a) the severity of the incident and (b) the capacity of the individuals involved to manage the situation without additional intervention. Evidently, those who believed that victims (50) or other bystanders (49) would accept the responsibility and have the capacity to effectively resolve or handle the situation were more likely to detach themselves from the situation and remain as passive bystanders. Those who had the tendency to minimize the severity of a situation, however, did not consistently adopt the role of a passive bystander as a result. For instance, individuals consistently ignored the incident when they perceived the absence of physical aggression to mean that the situation was not severe enough (59), or because they perceived the inaction of other bystanders as a sign that there was no need for intervention (49). On the other hand, while some individuals remained passive when they perceived the incident as being mere banter among peers (63), others had chosen to reinforce it (49, 63). This implies that there may be two stages in the process leading to a behavioral response, whereby the initial step involves factors that first influence the perception of the severity of the event, followed by the second step which includes an additional variable that subsequently influences the resulting type of negative cyber-bystander behavior.

An individual's perception regarding their responsibility to intervene, on the other hand, was in many instances associated with the nature of their relationship with participants involved in the incident, as individuals only felt more responsible to intervene when they were close to the victims (44, 55), hence were less likely to intervene when they had neither a close nor positive relationship with the victim (43, 46, 50, 55) or any other participant in general (44, 63). Moreover, when faced with ambiguous situations that make it difficult for individuals to even identify whether intervention is necessary, a close relationship with other participants would allow them to directly request for further context or clarification, and subsequently determine whether they must or want to intervene (63). Whereas, those with a weaker relationship with other participants would be deprived of this opportunity. Given the lack of contextual information present online, and the fact that the ambiguity of a situation leads individuals to remain passive, this presents as a vital finding (43, 63).

Regarding the lack of skills that were associated with negative bystander behavior, as mentioned in the result section, both the lack of problem-solving (52) as well as socio-emotional skills (47) resulted in negative cyber-bystander behavior. Similarly, factors like the lack of self-reliance (52), defending self-efficacy (50, 54), general self-efficacy (43), social self-efficacy (55) and components of empathy (43, 47), which are associated with these skills, were also associated with an increased likelihood that individuals would resort to negative cyber-bystander behavior. Building these skills and improving one's self-efficacy, which could promote later positive cyber-bystander behavior, requires both, the opportunity to learn and to practice those skills. One of the reasons that hinder these possibilities is the reliance on others, like authority figures, when faced with incidents of cyber-aggression, rather than relying on themselves, as it would likely decrease the opportunities to build these necessary intervention skills (59). Therefore, although teaching individuals to reach out to authorities or others who may be able to help or guide them is necessary, it is also important to create opportunities for them to develop these crucial skills.

Moreover, one's environment needs to promote these behaviors as being desirable behavior in order to further encourage it as the review indicates that individuals tend to engage in these behaviors if they believe that their peers expect (28) or will reward such behaviors (52), or that it would keep them safe (43, 49–51). This includes efforts to discourage both face to face and online cyber-aggression as those who were in classrooms with a higher number of aggressors were more likely to reinforce aggression (40). In addition to that, efforts to improve poor peer relationships and peer support also seem vital as factors such as self-efficacy were found to be associated with these variables (51), and it might also increase one's sense of safety when they have adequate social support. Based on the above-mentioned section, it also seems reasonable to believe that good peer relationships have the potential to promote a higher sense of responsibility (44) to engage in incidents of aggression as constructive cyber-bystanders.

It shouldn't go unnoticed that some individuals who lack defending self-efficacy may still assist the victim, albeit resorting to aggressive behaviors directed at the aggressor (54). This indicates that some individuals may have the correct intention, the ability to understand that intervention is required, and subsequently choose to intervene, but choose retaliatory acts similar to that of cyber-aggressors rather than more constructive ways of intervening. Schultze-Krumbholz et al. (47) identified that factors such as past experience as either a cyber-victims or cyber-aggressors, lower socio-emotional skills, as well as higher reactive-aggression, and possibly also impulsivity as being associated with these individuals. Bussey et al. (54) and Luo and Bussey (57) on the other hand, caution that these individuals are more closely linked to aggressors rather than defenders, making it necessary to clearly differentiate the various types of negative cyber-bystander behavior rather than grouping them together.

Another important theme that was identified through the review was the fact that there seemed to be notable distinctions between factors associated with aggressive and passive types of negative cyber-bystander behavior, despite there being some overlapping variables. For instance, aggression, whether it's their own past experience as aggressors (28, 42–44), increased exposure to aggressors in their environment (40), or their belief that aggression can be a valid response (53), was naturally more likely to be associated with aggressive forms of cyber-bystander behavior. Moreover, these behaviors were also associated with the belief that it would result in favorable responses such as an improvement in social status or opportunity for new friendships (52). On the other hand, passive bystanders were associated with factors which were related to avoidance of undesirable consequences like a threat to their safety (48), potential victimization (43, 49–51), loss of friendships (43) and etc. As highlighted above, it also has to do with not accepting responsibility to intervene, as well. However, this should be interpreted with caution given that studies had rarely addressed or compared the different types of negative cyber-bystander behavior.

In addition to above-mentioned themes, moral disengagement strategies, whether or not explicitly studied, were observed to be present in many of the situations that were discussed in the findings of the study. Simply put, moral disengagement is a mechanism of thought through which individuals rationalize and justify their harmful or generally negative behaviors (56), which in the case of the study would subsequently lead to or is simply positively associated with negative cyber-bystander behavior (49, 54, 57, 58). It involves the use of strategies such as the attribution of blame, the displacement or diffusion of responsibility, cognitive restructuring, distorting consequences of an action and etc. (56), most of which can be identified within the current review even in studies outside of those that had provided empirical evidence pertaining to their relationship with negative cyber-bystander behavior.

Some of the examples include the diffusion of responsibility experienced by individuals in a large crowd (60), placing blame and the responsibility to handle the situation on the victim (43), detaching themselves from their aggressive friends and the consequences of their behaviors (64), trivializing their reinforcement of aggression and reframing it as mere banter (49) or just the minimization of the severity of the incident in general (43), among other things. Moreover, aside from reinforcer and passive bystanders, both empirical evidence and simple deduction suggest that aggressive defending, was associated with moral disengagement strategies (54, 57), as it requires individuals to justify why their use of aggression is morally correct while the aggressor's use of aggression was not. These findings suggest that individuals resort to moral disengagement strategies in order to simultaneously maintain their own moral identities while also engaging in negative cyber-bystander behavior without feelings such as guilt. Additionally, moral disengagement was found to moderate the relationship between gender and pro-aggressive bystander behavior, further implying that it might be the factor that distinguishes between individuals that resort to such behaviors and those who don't (47).

Similarly, the bystander effect, as introduced by Latane and Darley (62), which suggests that individuals tend to resort to being passive in the presence of other bystanders (60), was proven by Chan et al. (49) and You and Lee (60) but contradicted by Song and Oh (64). This indicates that even the presence or absence of other bystanders did not consistently predict the way in which individuals will react to an incident of cyber-aggression, as some bystanders were more constructive in private situations where no or very few bystanders were present (64) while others were more likely to remain passive in a similar situation (42), and some others were passive in the presence, not absence of other bystanders (60). Therefore, it seems likely that a larger crowd, in some situations would increase the social pressure an individual experiences to behave in socially acceptable ways (42), while it encourages the diffusion of responsibility in others (60), and that other contextual factors should be taken into account. For instance, individuals with a close relationship with the aggressor were more likely to remain passive in the absence of other cyber-bystanders but were more likely to behave constructively in the presence of other bystanders (64), possibly because they found safety in numbers.

Lastly, although this phenomenon is one that occurs in cyber-space, there were very few papers which identified or acknowledged the influence of the characteristics of cyber-space and computer mediated communication on leading individuals to resort to negative cyber-bystander behavior. From what was reported, however, it can be gathered that the lack of context cues can complicate matters relating to the interpretation of the event and subsequently one's response as a cyber-bystander. Additionally, it seems as though individuals have the tendency to perceive cyber-aggression as being less severe than face-to-face aggression, solely due to the absence of physical aggression in the former, suggesting that there is a lack of awareness regarding the effects of cyber-aggression due to these differences. Moreover, asynchronicity which can cause a delay between when the incident had occurred and when the cyber-bystander witnesses it may leave individuals believing that there is no other response other than ignoring the incident as there is neither a need nor value in intervening in a situation that has already passed and might have been resolved (43). Besides that, in You and Lee (60), it was discussed that individuals are less likely to choose more constructive cyber-bystanders behavior, likely due to the fact that they fear negative judgments a lot less in such situations. Other than that, there seems to be very little empirical evidence and the discussion it fueled, regarding the ways in which the characteristics of cyberspace and the pattern of communication in such platforms influence negative cyber-bystander behavior.

### Future studies

Based on the review of demographic factors associated with the participants included in the studies reviewed, it is evident that the focus was largely on the pre-teen and adolescent population, aged between 12 and 16, with only six out of 27 studies addressing the adult population, and even fewer studies which included children ages 8–10. Hence, future studies should consider investigating negative cyber-bystander behavior among the adult population as well as well as younger children considering that the age at which individuals are exposed to the internet and technological devices seems to be decreasing. Moreover, various factors such as empathy, technological savviness, the need for external guidance and others might manifest differently among different age groups.

Secondly, most of the studies were carried out in the United States of America (USA) or European countries with very few addressing countries from other regions with differing cultures and norms. Given that these differences can subsequently influence thought and behavioral outcomes, it seems necessary to widen the scope of the study in terms of geographical locations, to account for cross-cultural differences. Additionally, most studies were cross-sectional surveys, more longitudinal studies as well experimental designs should be explored. In addition to that, there was a great benefit in collecting qualitative data from participants, hence, this practice should be encouraged even in the smallest ways in future studies.

Future studies could also go beyond the biological binary when discussion gender differences and account for more personal differences. The differences can include factors such as normative beliefs associated with gender, particularly with regards to aggression, empathy, outcome expectations, feelings of guilt and shame associated with negative cyber-bystander behavior, and the like. Moreover, difference in personality traits may also be worth exploring to identify variables that directly or indirectly influence an individual's choice of negative cyber-bystander behavior on a more personal level. In addition to that, factors such as moral disengagement, on the other hand, could potentially prove to be a vital moderating factor in future studies.

Future studies could also benefit from a more uniform way of measuring different types of cyber-bystander behavior to ensure some consistency across different studies. Most importantly, as identified above, more studies should address the ways in which the characteristics of cyberspace as well as computer mediated communication would influence an individual's decision to resort to negative cyber-bystander behavior. Various existing models and theories such as the online disinhibition theory by Suler (66) and the Barlett and Gentile Cyberbullying Model would be of use in doing so as they do indeed support findings presented by studies such as You and Lee (60) and Van Cleemput et al. (43).

### Limitations

There are a number of limitations and shortcomings in several aspects of the study ranging from the literature search, data analysis to the determination of the quality of the study. Firstly, although the choice of keywords used, as well as the strict inclusion criteria were selected and employed in order to ensure that only relevant data would be retrieved, it could have inadvertently excluded other significant literature which could have further enriched the current review. For instance, while the peer-reviewed articles published in the past ten (10) years might be able to better capture dynamics of the phenomenon in the current cyber landscape, it is likely that literature published prior to the past decade might contain important information that the current review may have benefitted from. The omission of gray literature would have impacted the review in similar ways. Moreover, limiting the literature search to five databases might have lead to the omission of important literature that could be found on other online databases or registers. Lastly, it should be taken into account the screening process could have been impacted by human errors. However, the review has managed to fulfill the aim of compiling, presenting and discussing important findings that provide a simple overview of negative cyber-bystander behavior.

Moreover, the reproducibility or the replicability of the search can be affected by things out of the control of the authors, such as the changes made in the literature databases in terms of search retrieval systems, the addition or elimination of journals or articles and etc. (67). Additionally, the quality of the review is difficult to assess given that the extraction and analysis of data could be influenced by bias as they rely on the reviewers' and authors' interpretations and ideas, and the fact that the review consists of studies of varying designs (68).

Despite the flaws, the review, like any other has managed to compile, and present a comprehensive set of literature and discussion highlighting factors associated with negative bystander behavior, in addition to the possible interactions between them, as well as possible gaps to address in the future. Moreover, the review can be used as a point of reference through which further questions can be identified, in order to extend the scope of research.

## Conclusions

Through the synthesis of literature included in this review, it is evident that is not only crucial to create an environment that facilitates and encourages positive bystander behavior, but also an environment that discourages and disincentivizes negative bystander behavior. This is especially true in the case of aggressive bystander behavior, as it would aid in not just preventing the reinforcement and/or exacerbation of an act of aggression by primary aggressors but could possibly prevent bystanders' potential future participation in acts of cyber-aggression. Moreover, the review indicates the necessity to take into consideration and further study in detail the interaction between multiple variables, as well as contextual factors, as a catalyst for negative bystander behavior as many of the studies have either theorized or proven that these are relevant in uncovering a clearer picture regarding this phenomenon. Lastly, the influence of technology on human behavior and interaction, in addition to the role of personal characteristics rather than a categorical approach to demographic differences may also prove to be useful directions for future studies to take.

## Data availability statement

The original contributions presented in the study are included in the article/Supplementary material, further inquiries can be directed to the corresponding author/s.

## Author contributions

The study and its design were conceptualized by SJ and MK. The literature search was carried out in two steps, whereby the preliminary screening was conducted by SJ and the subsequent screening to select studies to be included was a joint effort between SJ, MK, SM, JK, and AA. Data organization and analysis, in addition to the writing of the first draft was carried out by SJ under the direction and supervision of MK. Lastly, the collective effort and agreement of all authors were involved in the process of proofreading and editing of subsequent drafts, as well as the approval of the final submitted manuscript.

## Conflict of interest

The authors declare that the research was conducted in the absence of any commercial or financial relationships that could be construed as a potential conflict of interest.

## Publisher's note

All claims expressed in this article are solely those of the authors and do not necessarily represent those of their affiliated organizations, or those of the publisher, the editors and the reviewers. Any product that may be evaluated in this article, or claim that may be made by its manufacturer, is not guaranteed or endorsed by the publisher.
